# ‘Everything is data’: towards one big data ecosystem using multiple sources of data on higher education in Indonesia

**DOI:** 10.1186/s40537-022-00639-7

**Published:** 2022-07-14

**Authors:** Ariana Yunita, Harry B. Santoso, Zainal A. Hasibuan

**Affiliations:** 1 Faculty of Computer Science, Universitas Indonesia, Depok, Jawa Barat 16424 Indonesia; 2 Faculty of Science and Computer Science, Universitas Pertamina, South Jakarta, 12220 Indonesia; 3 Faculty of Computer Science, Universitas Dian Nuswantoro, Semarang, Jawa Tengah 50131 Indonesia

**Keywords:** Big data, Higher education, Data collection, Data preprocessing, Indonesia

## Abstract

Big data is increasingly being promoted as a game changer for the future of science, as the volume of data has exploded in recent years. Big data characterized, among others, the data comes from multiple sources, multi-format, comply to 5-V’s in nature (value, volume, velocity, variety, and veracity). Big data also constitutes structured data, semi-structured data, and unstructured-data. These characteristics of big data formed “big data ecosystem” that have various active nodes involved. Regardless such complex characteristics of big data, the studies show that there exists inherent structure that can be very useful to provide meaningful solutions for various problems. One of the problems is anticipating proper action to students’ achievement. It is common practice that lecturer treat his/her class with “one-size-fits-all” policy and strategy. Whilst, the degree of students’ understanding, due to several factors, may not the same. Furthermore, it is often too late to take action to rescue the student’s achievement in trouble. This study attempted to gather all possible features involved from multiple data sources: national education databases, reports, webpages and so forth. The multiple data sources comprise data on undergraduate students from 13 provinces in Indonesia, including students’ academic histories, demographic profiles and socioeconomic backgrounds and institutional information (i.e. level of accreditation, programmes of study, type of university, geographical location). Gathered data is furthermore preprocessed using various techniques to overcome missing value, data categorisation, data consistency, data quality assurance, to produce relatively clean and sound big dataset. Principal component analysis (PCA) is employed in order to reduce dimensions of big dataset and furthermore use K-Means methods to reveal clusters (inherent structure) that may occur in that big dataset. There are 7 clusters suggested by K-Means analysis: 1. very low-risk students, 2. low-risk students, 3. moderate-risk students, 4. fluctuating-risk students, 5. high risk students, 6. very high-risk students and, 7. fail students. Among the clusters unreveal, (1) a gap between public universities and private universities across the three regions in Indonesia, (2) a gap between STEM and non-STEM programmes of study, (3) a gap between rural versus urban, (4) a gap of accreditation status, (5) a gap of quality human resources distribution, etc. Further study, we will use the characteristics of each cluster to predict students’ achievement based on students’ profiles, and provide solutions and interventions strategies for students to improve their likely success.

## Introduction

With the development of technology and communication in recent years, data have become increasingly heterogeneous, and their volume has increased exponentially. The prediction of global data growth, based on a report from IDC, from 33 Zettabytes (ZB) in 2018 to 175 ZB in 2025 [1]. This report was made prior to the COVID-19 pandemic, in which case the report became invalid. During a pandemic, of course, the amount of global data currently available will increase along with all lines of organization and industry implementing physical distancing and almost all business processes turning online. In addition, today, digital traces, locations and activities can be recorded on the internet. In other words, almost existing real entities have virtual copies.

Technological improvements have resulted in a deluge of data from various areas, including health care, education, social media, and internet use, among others. This phenomenon has led to a new term: big data. Big data, the data that comes from multiple sources, multi formats, and characterised by the ‘five Vs’ [2], has risen in popularity as a research area and has created numerous issues and opportunities for researchers. There are various issues arise as a result of digitalization that need to connect one source of data to another source of data, for example, the spread of COVID-19 disease that leads to the pandemic. The pandemic involves data from multiple sources, such as residential data, which belong to the Ministry of Home Affairs, patient’s data from the Ministry of Health and all hospitals in Indonesia, then data related to geospatial in Indonesia. In the meantime, overcoming the pandemic need all three data sources simultaneously, which are handled separately by each institution. Accordingly, the challenge is how to logically integrate those various sources of data into one big data source, so that managing the pandemic can be effective.

Another example, it is common that Supply Change Management (SCM) and Customer Relationship Management (CRM) might be separately managed [3, 4]. Consequently, there is a mismatch between supply and demand. The challenge is how to optimize supply and demand, by integrating consumer behavior, origins, and consumption patterns into the supply chain. CRM and SCM capabilities can help businesses improve their competitiveness [5], thus to optimize the analysis, both of data related to SCM and CRM should be integrated logically.

Other challenges and opportunities are to collect universal big data in order to generate patterns, reveal the hidden structure and gain insights that might be useful to a variety of stakeholders, and, ultimately, make decisions [6] in a variety of real-life applications and services, including health care [7], business [8], online learning [9, 10] and sociocultural contexts [11]. This study puts big data ideas into practice in the higher education system in Indonesia. One of the issues in higher education in Indonesia is anticipating appropriate responses to students' academic achievements. Lecturers frequently apply "one-size-fits-all" policies and strategies to their classes. However, due to a variety of reasons, the level of understanding among students may differ. Furthermore, it is frequently too late to intervene to save a student's failing grades.

This study gathered data from students in Indonesia who had distinct characteristics. Indonesia is a middle-income country with a population of 273 million [12] and 300 ethnics spread across 17,744 islands [13]. Indonesia also has around five thousand higher education with various types of institutions. Furthermore, geographically, the regions in Indonesia also lead to the existence of digital divide [14], and the wide spectrum of infrastructure. In addition, socio economic conditions vary widely across the country. Predicting and prescribing intervention strategies for students in Indonesia remain challenging due to the country's characteristically diverse students.

Following the recommendations of previous research [15], this study built a big dataset that can be used for predictive and prescriptive analytics by collecting from multiple sources and integrating logically. In addition, the issue of ‘one data’ for Indonesia, which is integrating all data, as a constitutional mandate [16] is another motivation for this research. Furthermore, to take advantage of the era of big data and enrich the diversity of existing datasets for education-related data mining [15], this study built a large dataset for the higher education ecosystem in Indonesia and preprocessed the data to ensure that they were ready for use in later stages. Various techniques were carried out to obtain insights and reveal the hidden structure, including feature correlational analysis, Principal Component Analysis (PCA) and clustering analysis.

The term ‘big data ecosystem’ is used in this study. Ecosystem or ecological system is a theory that describe a geographical area where plants, animals, and other organisms, as well as weather and landscape, function together to form a bubble of life [17, 18]. In the ecosystem there is a circular activity, which can be defined what kind of entities involved in it. Referring to the theory of relational databases in one organization, data at an institution, such as the Ministry of Home Affairs can be related to external relational databases, such as the Higher Education Database. By defining big data is universal, it means that the data covering all entities in the ecosystem without exception. Big data ecosystem involves data from multiple sources that connect each other and support for further analysis such as gaining insights.

This study attempts to, first, propose a method to collect and preprocess data from multiple sources in order to form a big data ecosystem that might be implemented in other cases; second, cluster students in a big data ecosystem so in advanced students in Indonesia might be analysed from various factors, such as regional factor and social economy factors. A student who lives in a certain geographic area and has a specific economic background, for example, need a personalized advice.

The paper is organized as follows. Section two will discuss the literature review, including the literature of big data, data collection and data preprocessing. The next section will provide the methods. Another section will explain the results and discussion. The final section will present the conclusion, and future research.

## Related work and theoretical foundations

This section outlines the literature relevant to the study of big data in the higher education context and the theoretical background of data preprocessing for education-related big data and PCA.

### Big data in the higher education context

While the term ‘big data’ is popular today, there is still debate over its precise definition. One aspect of this debate is what size qualifies a dataset as ‘big’: what is ‘big’ today is likely to become comparatively small in the future. In addition, there are various points of view on large-scale data. One perspective from professionals in the field of data analysis is that big data refers to the process of extracting, transforming and loading large data [2]. Although there is no consensus on how big data should be defined, some literature claims that big data is synonymous with its V characteristics, namely Volume, Variety and Velocity. The characteristics of big data were first defined by Douglas Laney, namely 3 V, then by IBM to 4 V, continued to 5 V by Yuri Demchenko, 6 V by Microsoft until now to be 9 V or 3^2^ V which has a hierarchical structure [19].

Despite the evolution of big data characteristics from 3 to 9 V, 5 V characteristics seems to be the most widely used in big data terms [19, 20]. The first V, namely volume, means a large amount of data and is usually in the form of log data or time series. The second characteristic is velocity which shows the speed of the data. Veracity indicates that the data may be dirty, whereas variety means that the data type may be structured, semi structured and unstructured. Finally, value indicates that big data holds potential value [21]. From the explanation of the V’s characteristics, the big data acquires data from a variety of sources and a variety of formats.

The use of big data in the higher education context cannot be separated from learning analytics and education data mining. In previous research, we conducted a thorough review of the use of big data for learning analytics and education data mining [15]. Most previous research has attempted to use big data to predict specific issues related to students, such as identifying future dropouts or at-risk students [22], grades [23], academic performance [24–26] and academic achievement [27].

Yang et al. [25] used learning activity datasets from edX and Maple T.A. to predict students’ academic success; given the substantial correlations among dataset features, PCA was used in that work rather than multiple linear regression. However, the proposed model does not appear to be applicable to other courses with a variety of learning activities. Similarly, another study used student learning logs from the Hellenic Open University to calculate the risk that a student would drop out [22]. That study proposed a framework for predicting students’ attrition, which yields the probability of dropout, but the system in use was still not fully automated. Lemay and Doleck [23] used students’ behaviour to predict academic achievement. Unlike [22] and [25], they predicted students’ grades on assignments based on their individual video-watching behaviour in a MOOC. Using classifier algorithms, they trained a model on video logs from two courses containing 6,241 instances. The limitation of that study was that the analysis was applied only to a single MOOC. In contrast, [24] used three separate university datasets and leveraged big data in learning analytics to boost student retention. When using big data from diverse sources, however, certain problems may arise, such as the likelihood of different grading procedures at each university, which could lead to bias. Qu et al. [27] used a layer-supervised multi-layer perceptron model to capture an altogether different sort of data: student consumption time and web login activity.

To summarise, each study used a distinct method in terms of data to predict students’ academic progress utilising big data in learning analytics. This study collected data from multiple sources related to students, integrated those data into a large dataset and preprocessed that dataset so that it could be analysed using descriptive, predictive and prescriptive analysis.

### Data preprocessing for education-related big data

Preprocessing aims to convert raw data into a clean and usable dataset. Some literature suggests that pre-processing contains several stages, such as handling of missing values, data integration, data transformation, and data reduction [28, 29]. One of the most important issues in data preprocessing is how numerical data are discretised, as several discretisers exist. Moreover, educational data are characterised by a high level of missing values; as a result, preprocessing large educational data is unique and requires specific steps. Data quality encompasses many issues, including incomplete data, unbalanced data, and inconsistent data.

Previous studies have attempted to review and apply several preprocessing techniques, since its effects on accuracy vary [30–32]. Using 25 datasets available in the UCI repository, [30] compared eight discretisers: ChiMerge, Chi2, modified Chi2, extended Chi2, class-attribute interdependence maximisation, class-attribute contingency coefficient (CACC), Ameva (an autonomous discretisation algorithm) and minimum description length principle (MDLP). The discrete datasets were modelled using five classifier algorithms: support vector machine, *k*-nearest neighbours, naïve Bayes, neural network and C4.5. The results of their study indicated three top discretisers for researchers: MDLP, Chi2 and CACC. Furthermore, they recommended using C4.5 as a classifier with MDLP or Chi2 as a discretiser.

Similar to [30, 31] compared several discretisers, focusing on three. Unlike [30], however, they used only one dataset—specifically, an education dataset. Based on their experiments, they proposed using unsupervised discretisation techniques with a histogram distribution, commonly known as equal-width binning, and the SMOTE oversampling technique to improve the accuracy of education datasets.

In a study focusing on not only discretisation methods but also other preprocessing techniques, [32] attempted to find an optimal combination of feature selection techniques and data discretisation, since the two processes are both essential in data preprocessing. They determined what combinations should be used to achieve the highest accuracy. Three types of feature selection techniques (filter, wrapper and embedded) combined with unsupervised and supervised discretisation were tested in an attempt to achieve the highest accuracy. They used 10 datasets provided by UCI, then applied several combinations of feature selection and data discretisation.

In sum, the choice of data preprocessing techniques varies and may affect accuracy. The selection of preprocessing techniques seems to be an art rather than a science.

### Principal component analysis (PCA)

Analysing large datasets can be expensive due to heavy computation requirements. Therefore, data reduction is an essential stage of data preprocessing. Reducing data can be handled in two ways: (1) reducing the population and (2) reducing the number of features [28, 29].

Reducing population, also known as numerosity reduction, refers to minimising the number of records, commonly using two types of methods: parametric and non-parametric [29]. Other methods that can be used to reduce number of rows include histograms and clustering. However, in the case of big data analysis, sampling is not suggested. Reducing the number of features includes two stages: feature extraction and feature selection. In recent literature [25], PCA has been used to reduce dimensions. Therefore, dimensionality was reduced using PCA in this work as well.

PCA is an algorithm for transforming data by extracting features, which the output are the linear combinations of the features. PCA produces the linear combination among features, then it might reveal the hidden structure of dataset. PCA transforms data into a new set of coordinates, wherein the greatest variance is captured on the first coordinate, the second greatest on the second coordinate and so on. The first coordinate is called the first principal component (PC), the second is called the second PC and so on. Each PC is a linear combination and orthogonal. The general algorithm for PCA [43, 44] is as follows:

- Centralise the data matrix *X*.
- Compute the covariance matrix using the following equation:
  $$C=\frac{1}{m}X{X}^{T}$$
- Calculate the eigenvalue and eigenvector using the following equations:
  $$A-\lambda I=0$$
  $$\left[A-\lambda I\right][X]=0$$
- Find a new variable (i.e. PC) by multiplying the original variable by the eigenvector matrix. The variance that can be explained by the new variable depends on the value of *ρI*.
  $$\rho I=\frac{{\lambda }_{i}}{{\sum }_{j=i}^{D}{\lambda }_{j}} \times 100\mathrm{\%}$$

Three methods can be used to determine the number of PCs for further analysis: choosing PCs with a total variance of at least 80%, choosing PCs with eigenvalues greater than 1 and analysing the scree plot to find the ‘elbow’ that has the steepest line in the plot.

**Fig. 1:**
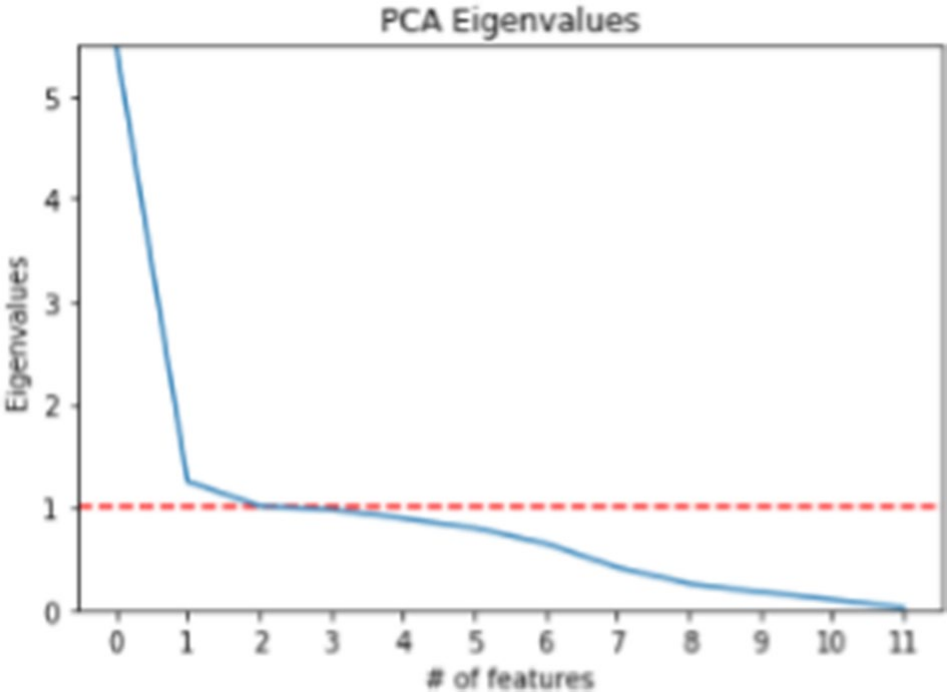
An example of scree plot of PCA Eigenvalues

For the plot shown in Fig. 1, there can be two or three PCs for two reasons. First, the eigenvalue is greater than 1 when the number of features is two or three. Second, when analysing the scree plot, the elbow occurs where the number of features is two or three. Thus, the total variance should be checked to determine whether two or three PCs should be used in further analysis.

In the following section, the research methods are explained.

## Methods

**Fig. 2:**
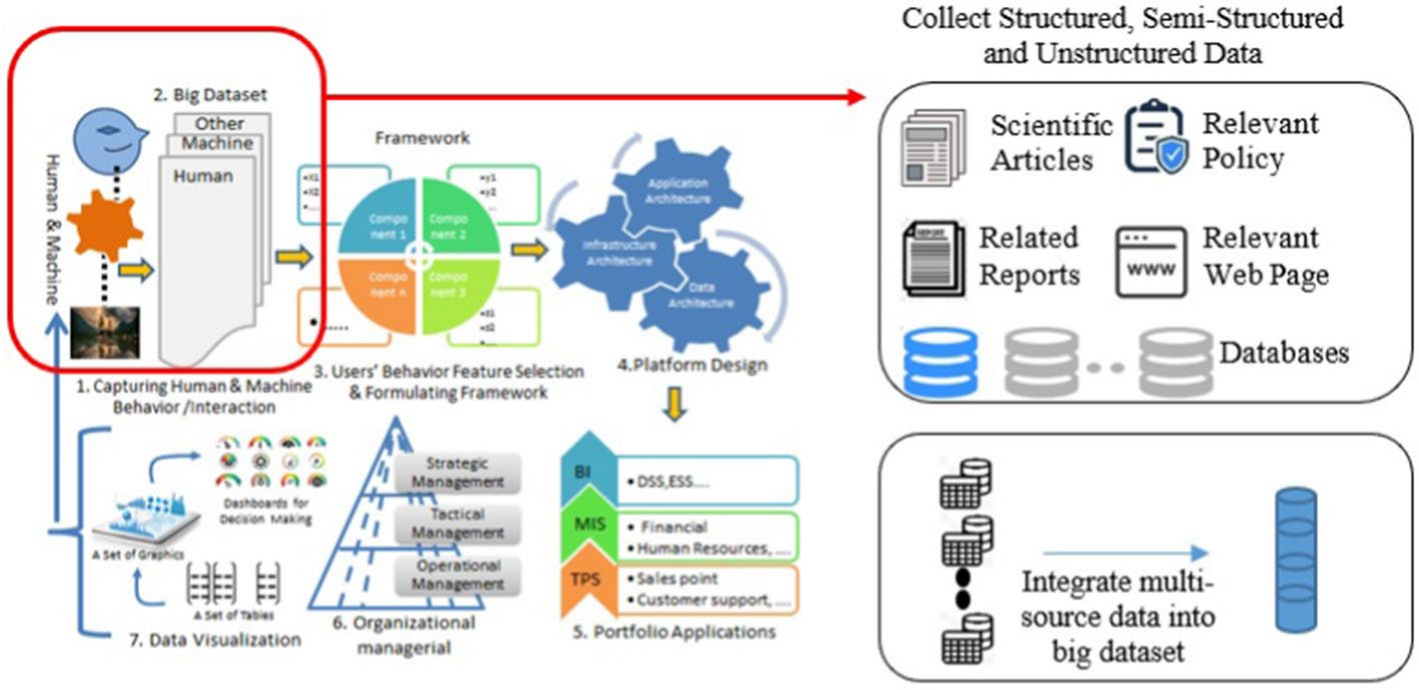
General research framework (modified from [6])

This study adopts the end-to-end life cycle of automation systems [6] as a general research framework. The main idea of this framework is that ‘everything is data’ and that those data can be collected and processed to identify patterns and produce meaningful insights that any stakeholder can use to make decisions. The end-to-end life cycle automation system bridges the two research paradigms in information system by taking advantage of research synergies between design science and [33] and behavioural science [34]. While previous study related to design science aims to develop new products or artifacts, and test whether these products are useful for their purposes [35], previous study related to behavioural science reveals the behaviour of humans or organizations, such as to find the correlation among factors or reveals factors contributing to the information system [34, 36, 37]. Thus, within the end-to-end life cycle automation system, in Fig. 2, the research phases 1 until 3 adopt behavioural science, while research phases 4 until 7 aim to develop a system. As visualised in Fig. 2, the first stage is collecting all possible data and the stage is building a big dataset. Below is an explanation of how we collected data from multiple sources and performed preprocessing to build a big dataset.

### Data collection from multiple sources

In Indonesia, student data are stored in a national database belonging to the Ministry of Education, Culture, Research, and Technology called the Higher Education Data Base (PDDIKTI). We collected data from various sources, including PDDIKTI, the National Accreditation Agency and the Central Statistics Agency. We used relevant policy, such as the Ministry of Education, Culture, Research, and Technology Regulation (in Bahasa, Permendikbud No. 3 of 2020), as our main reference for data consistency. Another source type used was relevant webpages, such as the website of the Ministry of Home Affairs, which we used to categorise data. Recent literature, such as [38], was used to categorise socioeconomic data. Related reports, such as the 2014 Indonesian Standard Occupational Classification (in Bahasa, ‘Klasifikasi Baku Jabatan Indonesia 2014’), were also used to categorise data.

Our rationale for selecting universities in Indonesia that were included in PDDIKTI was our aim of representing data on students in Indonesia. Several categories were included to develop a dataset that was representative of all conditions in Indonesia, such as type of university (public or private), accreditation status and location. Other considerations included:

- Type of higher education institution selected (i.e. a university)
- Five universities each selected from the western, central and eastern regions of Indonesia
- Date of the Higher Education Decree chosen was at least 2013 so that a minimum of three batches were taken
- Student data retrieved from 2011 through the end of 2019 (before the COVID-19 pandemic)

**Fig. 3:**
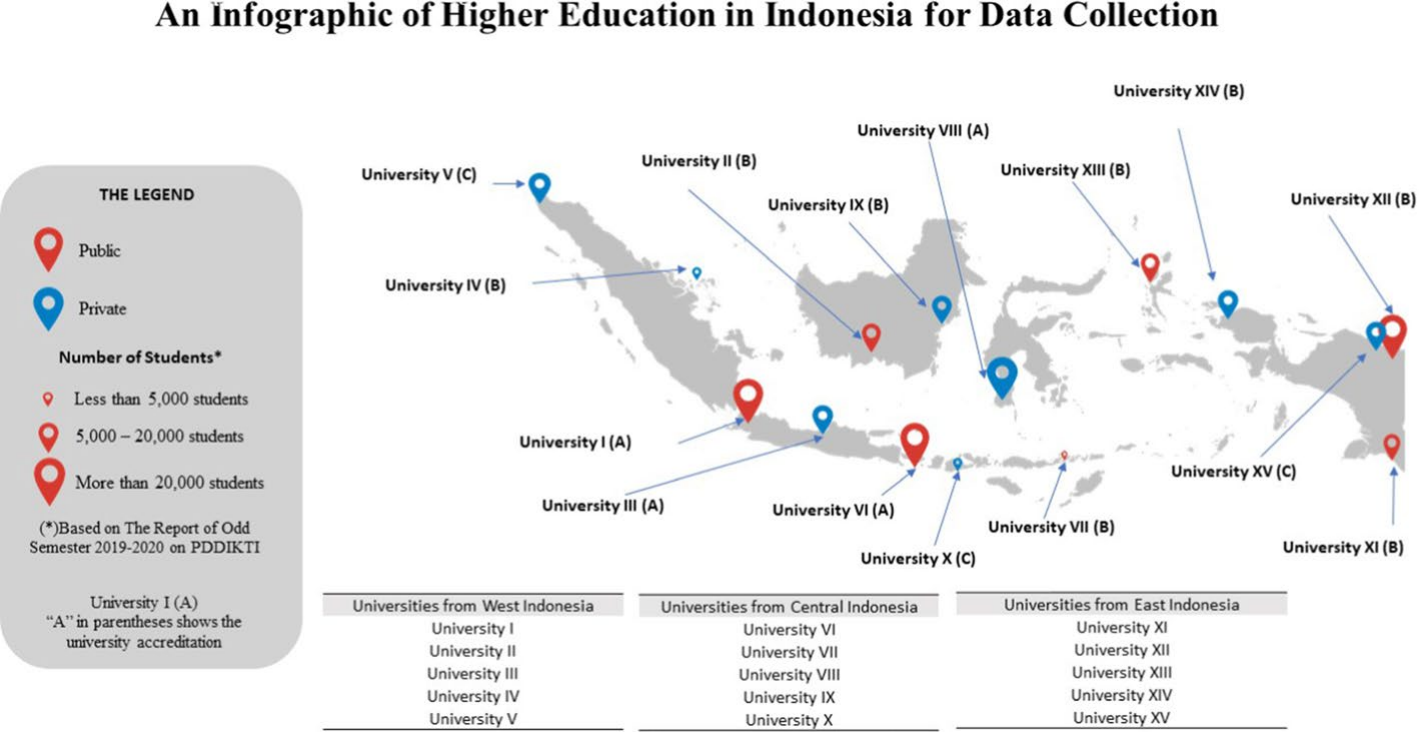
Infographic representing collection of data on higher education in Indonesia

Figure 3 shows information on 15 universities (modified for anonymity) collected for this study. Division of the study region into three subregions (western, central and eastern) follows the division of time in Indonesia. The western Indonesia region covers Java, Sumatra and half of Borneo (West Borneo and Central Borneo). The central Indonesia region covers Bali; North, East and South Kalimantan; Sulawesi; and West Nusa Tenggara to East Nusa Tenggara. The eastern Indonesia region covers Maluku, North Maluku, Papua and West Papua. It is important to note here the significant difference between eastern and western Indonesia. There are no A-accredited private or public universities in eastern Indonesia, and university VIII is the only A-accredited PTS outside the island of Java. Conversely, there are no C-accredited universities found in the western region.

The raw data obtained from PDDIKTI already included data that were planned to be taken at the National Accreditation Agency. Furthermore, we integrated the raw data received from PDDIKTI with data from other data sources, such as the Central Statistics Agency and the Ministry of Home Affairs.

The raw data contain 1,291,297 rows and 41 columns. The collected raw data were analysed to determine the number of unique values and the values contained in each column. Several columns contained no values, such as columns related to students’ achievements. Data related to socioeconomic background also contained many missing values.

Another issue was that the data were not yet organised such that there was a single row of data per student. Thus, it was necessary to generate pivot tables first. Data processing could not be performed with Microsoft Excel because the number of rows of data exceeded one million. Therefore, the processing of the raw data was performed in Python, using Google Colaboratory (‘Colab’) for the data integration stage. From the raw data that were integrated, students at the undergraduate level were selected for the subsequent process.

### Data preprocessing

First, we gained a broad understanding of the data by viewing a description of the data in the form of means, modes, medians and quartiles. In addition, the number of total missing values was calculated for each column to determine data quality. Afterward, preprocessing was performed drawing on relevant sources, one of which was the National Higher Education Standards (Permendikbud No. 3 of 2020). At this point, the raw data underwent various preprocessing techniques, including data categorisation, data consistency, data quality assurance and missing value replacement.

#### Data categorisation

Even though literature suggest many techniques for discretizers, we discretized by referring to other reliable and trustworthy data sources. For example, for the columns representing student academic history in the form of grade point average (GPA) each term, average GPA and number of credits, we referred to the National Standards of Higher Education. For the column representing the employment sector of students’ parents, we used as a reference the Job Classification in Indonesia document launched by the Central Bureau of Statistics. Types of programmes of study were classified as science, technology, engineering and mathematics (STEM) or non-STEM following the STEM and non-STEM grouping documents from the Ministry of Education and Culture.

For parents’ income (either father or mother), our groupings were based on reports from the Asian Development Bank [38]. Those reports sort by total daily consumption, which ranges from $2 to $4 for the lower class, also labelled the floating class. The next tier is those with total daily consumption ranging from $4 to $10, representing the upper-middle class. Those who spend $10 to $20 per day are categorised as upper-middle class and those who spend $20 to $100 per day are categorised as higher class. Based on the statement, we adjusted the classes created by the Asian Development Bank to fit our data, resulting in five classes: poor, very low (floating), low (lower middle), medium (upper-middle) and high (higher).

#### Ensuring data consistency

Data consistency was ensured based on the Higher Education Standards document contained in Permendikbud No. 3 of 2020. Several related stakeholders who had the same data were also used as references to check the consistency of the data. For example, the Ministry of Home Affairs classifies education levels for Family Card (in Bahasa, ‘Kartu Keluarga’) data.

Several problems were found, which became important to address in the data consistency stage. The first issue was the inconsistency between GPA each term, total GPA and number of credits recorded by PDDIKTI—for example, rows where the number of credits was 0 but the GPA each term and GPA values were filled. Rows where there was an inconsistency between GPA and number of credits were deleted.

The second issue involved graduates who had fewer than 144 credits. For example, some students were found to have completed only 142 credits. Likewise, with regard to the maximum number of academic years enrolled before graduation, we found that some students were able to graduate despite having been enrolled for more than eight academic years. Other issues were related to the names of provinces, cities, programmes of study and education levels. The names of provinces, cities and districts did not match the data from the Ministry of Home Affairs. Furthermore, the names of the programmes of study did not match the STEM versus non-STEM list. The names of parents’ education levels were adjusted to match the data from the Ministry of Home Affairs.

#### Removing outliers and handling missing values

Before eliminating outliers, data visualisation analysis was carried out using boxplots to identify the outliers in each column in the dataset. The results indicated that several values were outside the normal range. This can occur due to incorrect input (e.g. a GPA of 3.45 is input as 345). Likewise, values of 200 for number of credits were due to input errors.

To eliminate outliers, the Higher Education standard was used. GPA values above 4.0 were removed, and numbers of credits above 25 were deleted. If an eraser with the interquartile method were used, the data would be reduced by 90%—in other words, only 10% of the data would remain. To ensure data consistency, we used the Higher Education standard: values above 4.0 for GPA each term and total GPA were removed, and values for number of credits above 25 were also deleted. It is important to note here that there were many missing values in the data on socioeconomic background, which presented a problem in the next stage of the process. Missing socioeconomic data values were filled in as ‘unknown’.

To summarise the phases of data preprocessing, data integration was performed on the existing raw data. Data cleaning was then performed to ensure data quality—specifically, consistency and elimination of missing values. Following that, categorisation was completed, resulting in the dataset described in “Results and analysis” section.

## Experimental environment

To conduct experiments in this study, a Jupyter notebook (running Python) was used because Jupyter is an easily understandable open source tool for development and generating knowledge and insights through data analysis. The hardware and software were provided by the AI Center Laboratory, which is equipped with an NVIDIA DGX-1 system. Several data mining techniques were used, namely exploratory data analysis, PCA and the *k*-means algorithm. Details of the software, hardware and libraries used for data analysis are provided in Table 1.

**Table 1:** Experimental environment

Type	Details
Hardware	NVIDIA DGX-1 supercomputer
Software	Kubernetes, Python, Microsoft Excel, Anaconda, Jupyter Notebook
Libraries	pandas, NumPy, seaborn, matplotlib, PCA, k-means

## Results and analysis

### Indonesian higher education students dataset

Applying the various preprocessing techniques to the raw data resulted in a preprocessed dataset, the structure of which is shown in Table 2.

**Table 2:** Dataset structure before one-hot encoding

No	Feature	Category	Data type
1	GPA each term 1,2,3…*n*	Academic history	Ordinal
2	Total GPA term 1,2,3…*n*		Ordinal
3	Term 1,2,3…*n*		Ordinal
4	regional_university	Geographical location	Nominal
5	city_district_univ		Nominal
6	type_univ	Institutional background	Nominal
7	accreditation_univ		Ordinal
8	type_study_program		Nominal
9	accreditation_study_program		Ordinal
10	Gender	Demographic factors	Nominal
11	Nationality		Nominal
12	Province		Nominal
13	district_city		Nominal
14	Economy	Socioeconomic factors	Ordinal
15	edu_background_father		Nominal
16	job_father		Nominal
17	salary_father		Ordinal
18	edu_background_mother		Ordinal
19	job_mother		Nominal
20	salary_mother		Ordinal

The features in the dataset were organised into the following categories: academic history, geographical location of the institution, institutional background, student demographics, and student socioeconomic background. The study’s output at this phase was a preprocessed dataset ready for analysis.

### Correlation analysis and PCA

**Fig. 4:**
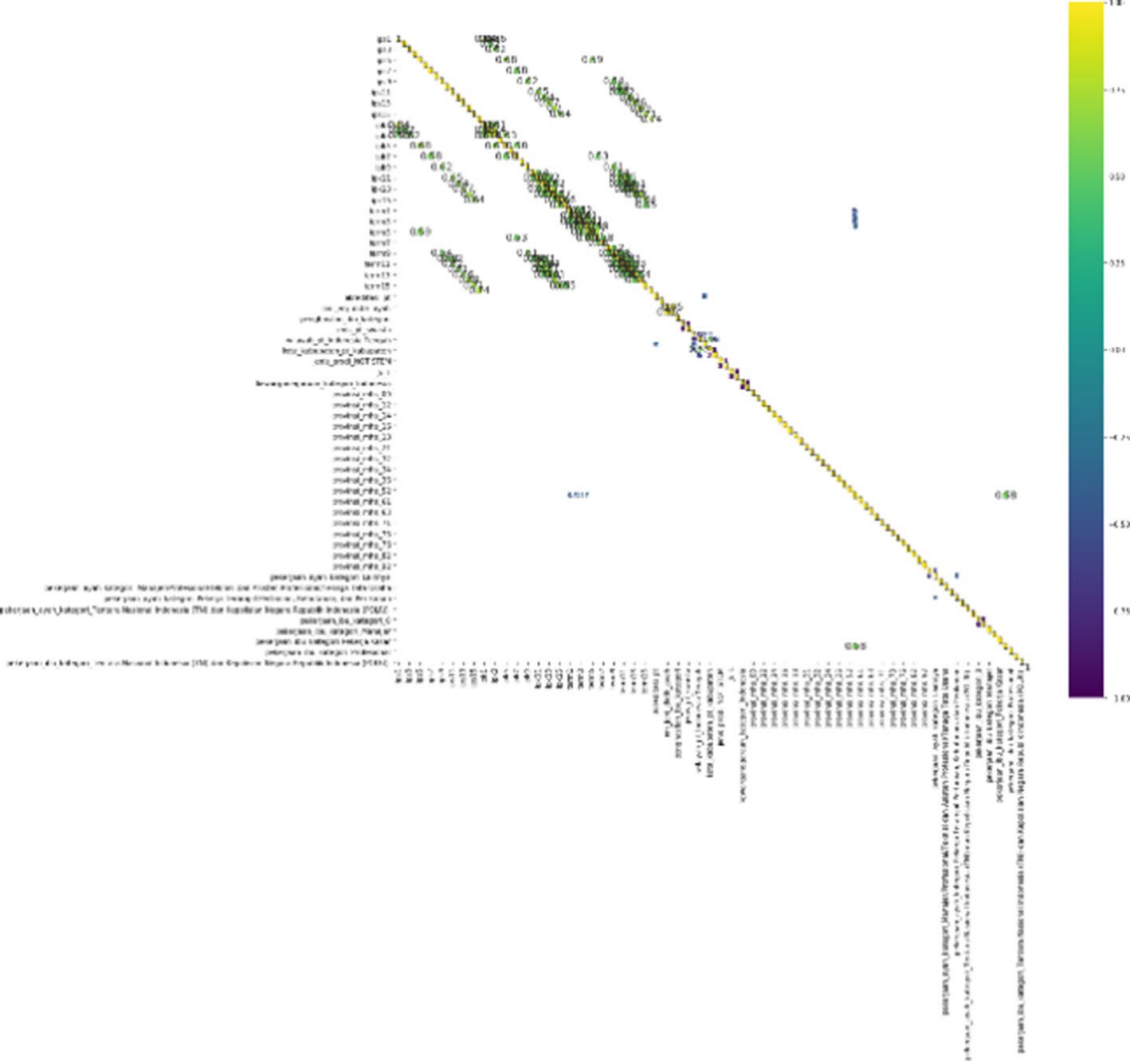
Feature correlation matrix

After preprocessing, data analysis was conducted using correlation analysis and PCA. Feature correlation analysis aims to identify the correlations of each feature so that feature selection or extraction can be applied. This assumes that the data are normally distributed. Matrix correlations (using Pearson’s correlation) for each feature are shown below. The correlation analysis below was conducted before performing data categorisation and one-hot encoding for categorical features (Fig. 4).

The results of the feature correlation matrix indicate that many columns or dimensions are correlated, suggesting that dimension reduction is needed. High dimensionality makes computation expensive and high value, so it is more effective to extract features using an algorithm. The algorithm chosen for this research was PCA.

**Fig. 5:**
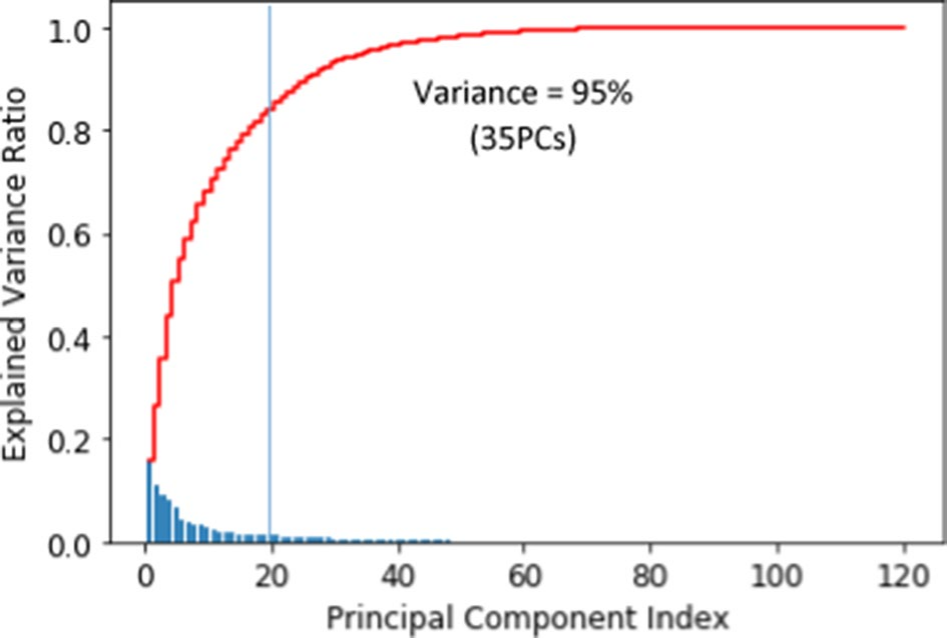
Principal component index

From the original 120 features, as shown in Fig. 5, 35 PCs were taken as linear combinations of the original features, capturing 95% of the variance. Interpretations of each PC are provided in Table 3.

**Table 3:** Feature representations for each principal component (truncated)

PC	Original feature representation	Label
PC1	west_Indonesia (0.17), study_program_STEM (0.13), gender_male (0.2)	West Indonesia, study programme STEM, gender male
PC2	private_univ (0.48), central_Indonesia (0.3), gender_male (0.25), study_program_STEM (0.2)	Private university
…	…	…
PC35	term8_cat (0.87)	term8

**Fig. 6:**
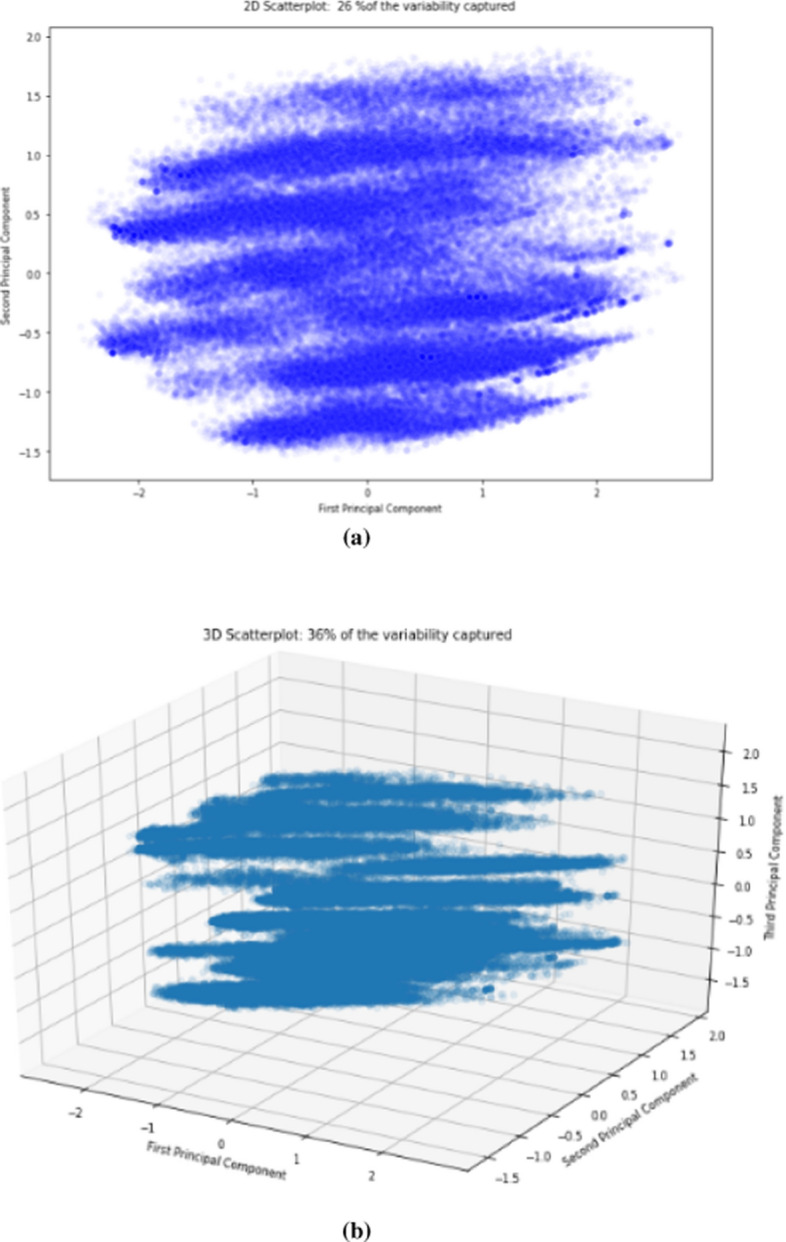
Two-dimensional (**a**) and three-dimensional (**b**) visualisations of the first two and three principal components

Next, two-dimensional and three-dimensional visualisations were performed for the first two and three PCs, respectively. The results of these visualisations are shown in Fig. 6.

The visualisations in Fig. 6 show an ellipse pattern, which merits further investigation. A label *Y* can be added to the dataset to see whether the pattern separates the *Y*. Next, clustering analysis of the 35 resulting PCs was conducted to understand the existing patterns.

### Clustering analysis

Clustering is the process of splitting a population or set of data points into many groups so that data points in the same group are more similar than data points in other groups. It aims to identify data points with similar characteristics and assign them to clusters [28]. Data can be divided into clusters based on centroids, distributions, densities or other factors.

**Fig. 7:**
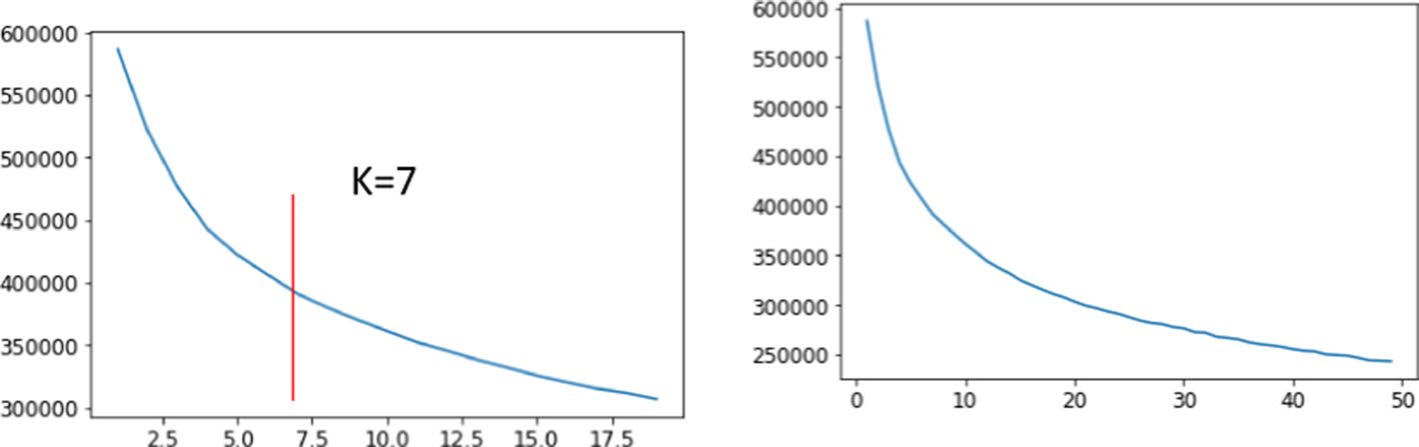
Elbow plots used to analyse values of *k*

The *k*-means algorithm clusters data based on centroids. This algorithm is well known for its simplicity and ability to cluster big data and outlier data very quickly. When using this clustering algorithm, each data must belong to a certain cluster. We applied *k*-means clustering to the dataset with 35 PCs. Several parameters can be used to determine the best number of clusters (i.e. best value of *k*), including the Davies–Bouldin index, silhouette value and elbow method. Various values of *k* were analysed using an elbow plot (Fig. 7). Different values of *k* were compared in the elbow plots by checking the error sum of squares value at the specified cluster.

Based on the elbow plot shown in Fig. 7, we chose seven clusters because the elbow is visible at that point. Afterward, we listed the numbers of data points in each cluster. Cluster 1 has the most data points compared with the other clusters, while Cluster 7 has the least. The exact number of data points in each cluster can be seen in Table 4.

**Table 4:** Number of data points in each cluster

Cluster	Number of data points
Cluster 1	19,895
Cluster 2	17,231
Cluster 3	16,593
Cluster 4	15,015
Cluster 5	14,886
Cluster 6	10,076
Cluster 7	7,534

**Fig. 8:**
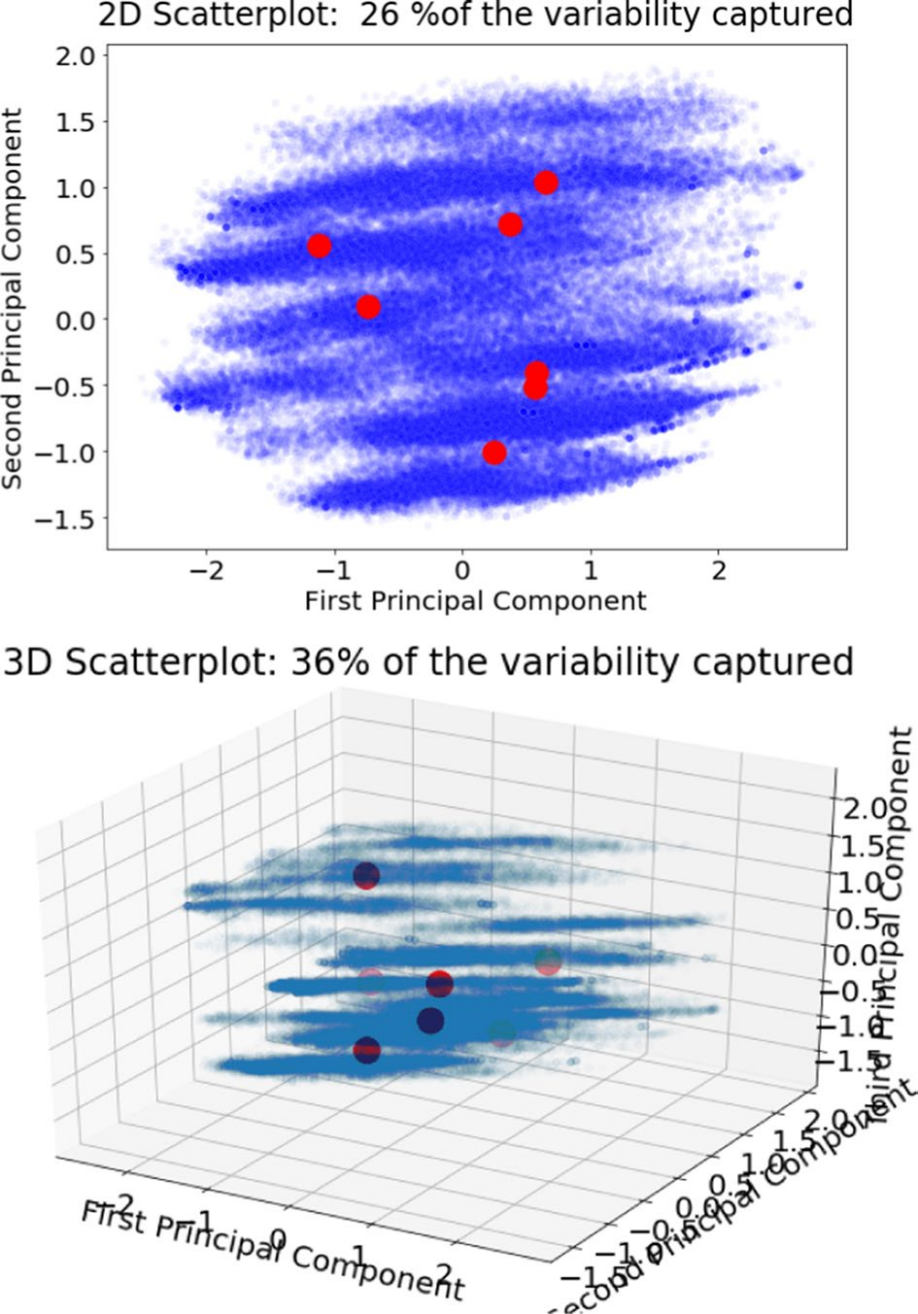
Two-dimensional and three-dimensional visualisations with centroids of seven clusters (*k*-means)

The next step was visualising the centroids of clusters using a two-dimensional visualisation of the first two PCs and a three-dimensional visualisation of the first three PCs (Fig. 8).

**Fig. 9:**
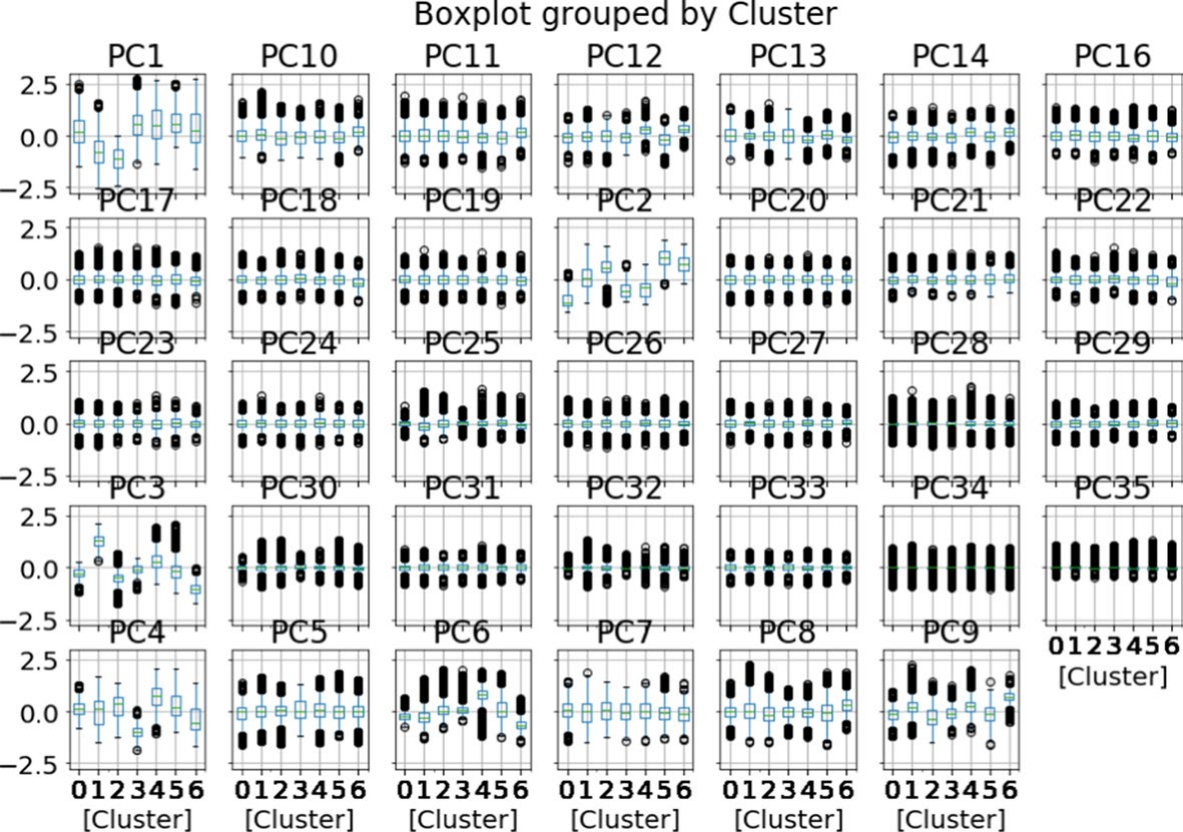
Boxplots grouped by cluster

The visualisations shown in Fig. 8 seem to indicate that the centroids did not follow the pattern of the visualisation. However, it was still difficult to analyse the clusters using two-dimensional and three-dimensional visualisations. The next step was analysing the characteristics of each cluster so that the clusters could be labelled. The process of labelling each cluster using boxplots is visualised in Fig. 9.

As shown in Fig. 9, several important PCs can differentiate each cluster, including PC1, PC2, PC3, PC4, PC6 and PC9. PC1 represents students who are male, attending a university in western Indonesia and enrolled in a STEM programme of study. As indicated in the boxplot analysis, Clusters 4, 5, 6 and 7 are relatively highly ranked for this PC. Cluster 1 is moderate, Cluster 3 is the lowest and Cluster 2 is the second lowest. PC2 represents students attending private universities. As can be seen in the boxplot analysis, PC2 can differentiate each cluster. Cluster 1 is the lowest ranked for PC2, while Cluster 6 is the highest, Cluster 7 is the second highest, Cluster 2 is moderate, Cluster 3 is relatively high and Clusters 5 and 6 are relatively low. PC3 represents students attending a public university in a district region, especially central Indonesia. For this PC, Clusters 1, 4, 5 and 6 are moderate, Cluster 2 is the highest and Cluster 7 is the lowest. PC4 represents students enrolled in non-STEM study programmes. Cluster 5 is the highest, followed by Cluster 5. Clusters 1, 4 and 6 have values that are almost zero, and Cluster 7 is the lowest. PC6 represents students attending university in eastern Indonesia. PC6 characterises each cluster, especially Cluster 5 (highest). Cluster 7 is the lowest. PC9 represents students in districts in eastern Indonesia and total terms within the first academic year. For this PC, Cluster 7 is the highest. Clusters 2 and 5 have values slightly above 0, and the values for Clusters 1, 3, 4 and 6 are very close to 0.

After analysing the characteristics of each cluster using boxplot analysis, the clusters can be interpreted as follows:

- Cluster 1: Public university students from various regions in Indonesia (western, eastern, central)
- Cluster 2: Public university students in districts in central Indonesia
- Cluster 3: Students in non-STEM programmes of study
- Cluster 4: Students in STEM programmes of study at a public university
- Cluster 5: Male students from western Indonesia in STEM programmes of study
- Cluster 6: Private university students from eastern Indonesia
- Cluster 7: Students in STEM programmes of study in districts in western Indonesia

**Fig. 10:**
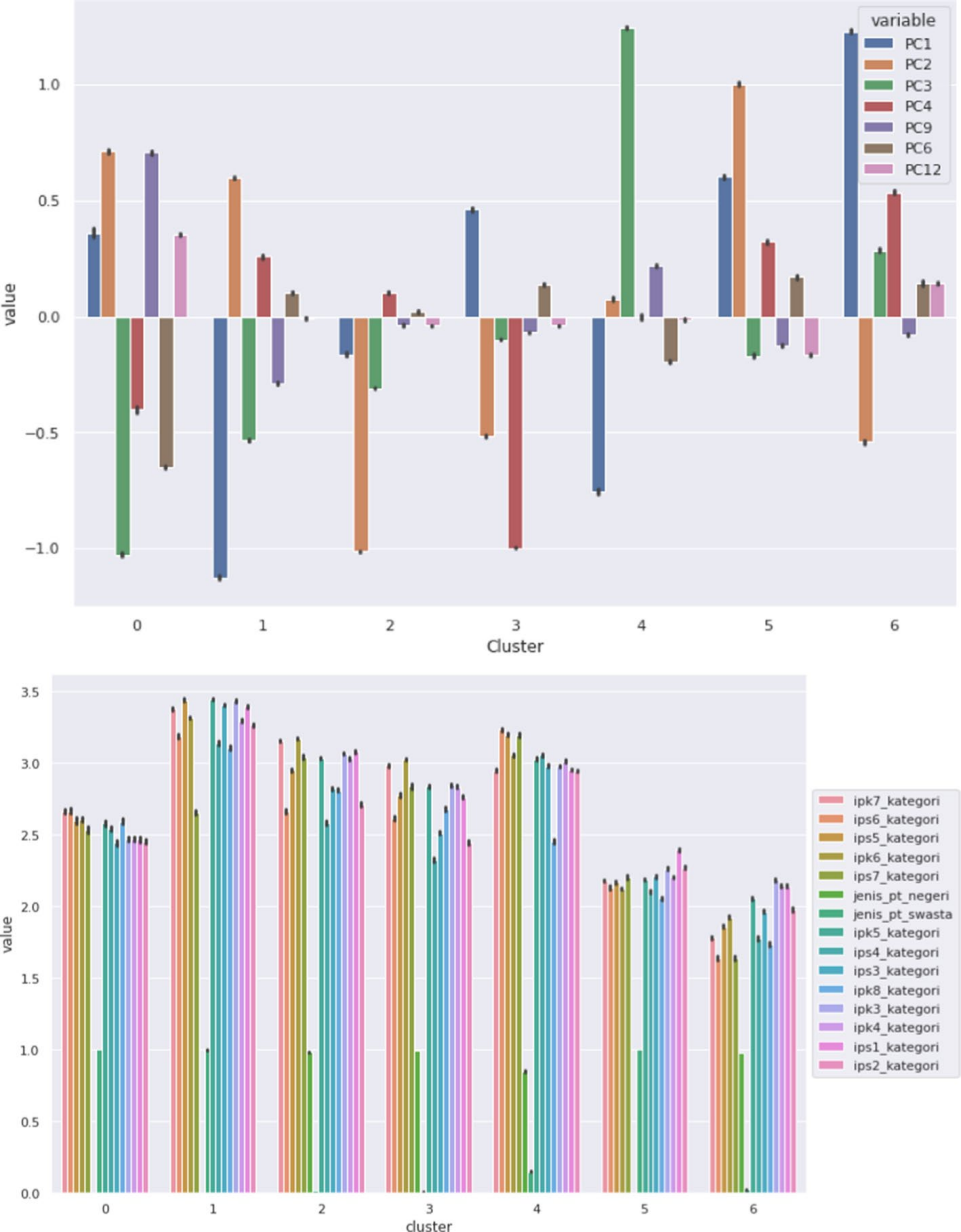
Bar plot analysis by cluster using **a** seven highest-variance PCs, **b** 15 highest-variance original features

Another method used to interpret the clusters was bar plot analysis. The variance of means between clusters within each variable was calculated, and the top seven variables with the highest variances were chosen. As can be seen in Fig. 9a, PC1, PC2, PC3, PC4, PC9, PC6 and PC12 were the seven PCs with the highest variance. Furthermore, the PCs were returned to the original features. A total of 15 features were selected. The results can be seen in Fig. 10b.

**Fig. 11:**
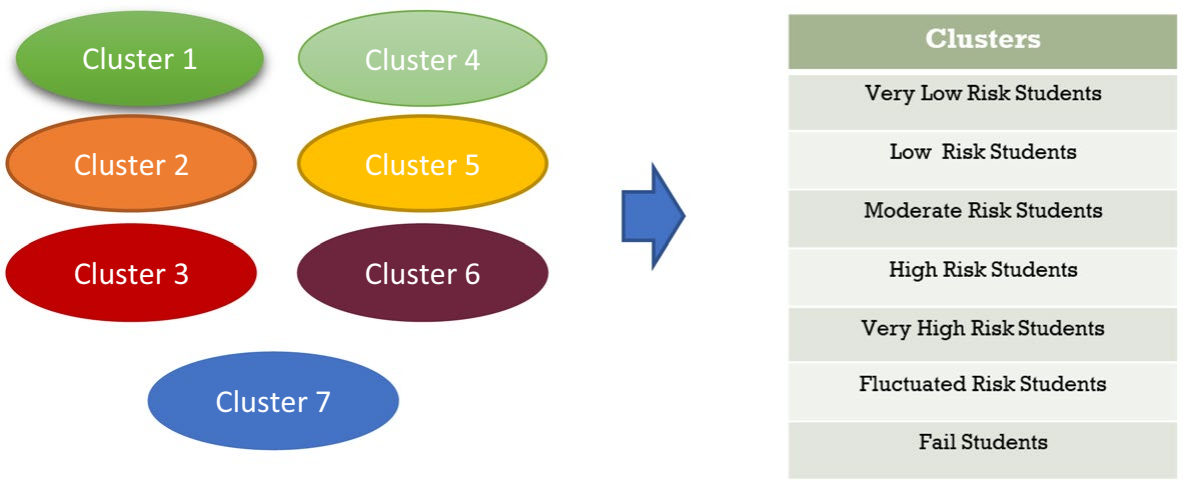
Labelling of clusters in dataset

As shown in Fig. 10b, GPA differentiates each cluster, and there is a gap between Clusters 1 and 7 in terms of GPA. Therefore, we labelled each cluster as follows: Cluster 0 as high-risk students, Cluster 1 as very low-risk students, Cluster 2 as low-risk students, Cluster 3 as moderate-risk students, Cluster 4 as fluctuating-risk students, Cluster 5 as very high-risk students and Cluster 6 as failing students. This labelling is visualised in Fig. 11.

Other features were also analysed by comparing each cluster, after which the clusters’ characteristics were retrieved. First, students in central Indonesia have lower GPAs compared with students in east and west Indonesia. Students at universities in districts tended to have lower GPAs than university students in cities. STEM students tended to have lower GPAs than non-STEM students. The clusters are defined by regions in Indonesia, location in district or city, university type (private vs public), and programme of study (STEM or non-STEM). Clusters 1 and 6 have the highest GPAs compared with the other clusters. Cluster 1 consists of students from public universities from various regions, while Cluster 6 consists of students from private universities and is dominated by students from eastern Indonesia.

The clustering analysis indicated a gap between public universities and private universities across the three regions in Indonesia. There was also a gap between rural and urban universities and between STEM and non-STEM programmes of study. Related to the issue of rural versus urban, several previous studies have shown that rural–urban gaps can affect learning [39, 40] due to inadequate infrastructures and a lack of high-quality teachers in rural areas. It is important to note here that rural–urban gaps might disappear if students had similar socioeconomic statuses.

There may be implications of this study related to infrastructure issues in Indonesia. The clustering might indicate a rural–urban gap in the conditions of the infrastructure of each region in Indonesia [14, 41]. According to [14], Jakarta has a low digital divide level, whereas the majority of Borneo and Java have moderate digital divide levels. Several provinces in Sumatra and Sulawesi also have moderate digital divide levels. Five of the 14 provinces with high digital divide levels are on the island of Sumatra, namely Aceh, North Sumatra, Jambi, Bengkulu and Riau Islands; two are on Java and its surrounding islands, namely Yogyakarta, Banten and Bali; and the remaining six are on Sulawesi, Maluku and Papua Island. It can be concluded that there is a high level of inequality in access to information and communication technology.

Another important issue is related to accreditation. Western Indonesia had no C-accredited universities, while eastern Indonesia had no A-accredited universities. Accreditation can be indicative of the quality of a university from the perspective of teaching, infrastructure and research activities. This might be in line with digital divide levels; therefore, further investigation is suggested for this issue.

According to Vygotsky’s learning theory of zone proximal development [42], which emphasises that knowledge is constructed through social interaction, there should be more research on the availability of competent lecturers in Indonesia and how this is related to students’ academic achievement. The distribution of professors in western, central and eastern Indonesia might have an impact on learning processes and outcomes. Our dataset could be supplemented with information about the background of the lecturers at each institution. In addition, future studies should validate and explore in greater depth the characteristics of students’ clusters and the relationships among clusters.

## Conclusions and recommendations

This study created a forerunner of the ‘one big data’ higher education ecosystem that includes a variety of data sources, such as national education databases, databases from other institutions, reports and webpages. This study generally followed the End-to-End Life Cycle Automation system’s research framework, in which the first step is to collect all possible data. Following the guideline ‘everything is data’, data were collected from a variety of sources, including PDDIKTI, scientific articles, reports and national policy. After being collected, the data were integrated and preprocessed. The study’s output is a relatively clean and sound big dataset that adheres to three rules: one column represents one feature; each student represents one row and each value has its own cell. Other preprocessing techniques can be applied to build the one big dataset, especially with regard to categorising data. PCA was used to minimize the dimensionality of a big dataset, while K-Means algorithm was used to reveal clusters (inherent structure) that may exist in the dataset. K-Means analysis has identified seven clusters: 1. very low-risk students, 2. low-risk students, 3. moderate-risk students, 4. fluctuating-risk students, 5. high-risk students, 6. very high-risk students, and 7. fail students. Clustering analysis also revealed that there was a gap between public and private universities across Indonesia's three regions, a gap between STEM and non-STEM programs of study, a gap between rural and urban, a gap of accreditation status, a gap of quality human resource distribution, and so on.

The research methodology in this study might be used for different research areas. Furthermore, the concept of big data ecosystem would strengthen the direction of new research in digital era. For future research, it is recommended to add more features related to the learning environment and to explore the clusters of students in greater depth. The clustering results can be used as preliminary results for predictive analytics and prescriptive recommendations for policy development, strategies and programme interventions to improve the quality of higher education. Our research agenda is also to add several features, such as the learning environment, lecturers’ publications, innovations and intellectual property rights at each institution. Moreover, the big dataset can be used to predict and give recommendations for several stakeholders, such as management at higher educational institutions, the central government (Ministry of Education and Culture, Higher Education Research and Technology) and so forth.

## Data Availability

The datasets generated and analysed during the current study are available from the corresponding author on reasonable request.
